# Genome Editing with AAV-BR1-CRISPR in Postnatal Mouse Brain Endothelial Cells

**DOI:** 10.7150/ijbs.64188

**Published:** 2022-01-01

**Authors:** Xiaopeng Song, Yaxiong Cui, Yanxiao Wang, Yizhe Zhang, Qi He, Zhenyang Yu, Chengfang Xu, Huimin Ning, Yuying Han, Yunting Cai, Xuan Cheng, Jian Wang, Yan Teng, Xiao Yang, Jun Wang

**Affiliations:** 1 State Key Laboratory of Proteomics, Beijing Proteome Research Center, National Center for Protein Sciences (Beijing), Beijing Institute of Lifeomics, Beijing 102206, China.; 2 Department of Immunology, College of Basic Medicine, Qingdao University, Qingdao, Shandong 266071, China.

**Keywords:** CRISPR/Cas9, brain endothelial cell, blood-brain barrier, genome editing, AAV-BR1

## Abstract

Brain endothelial cells (ECs) are an important component of the blood-brain barrier (BBB) and play key roles in restricting entrance of possible toxic components and pathogens into the brain. However, identifying endothelial genes that regulate BBB homeostasis remains a time-consuming process. Although somatic genome editing has emerged as a powerful tool for discovery of essential genes regulating tissue homeostasis, its application in brain ECs is yet to be demonstrated *in vivo.* Here, we used an adeno-associated virus targeting brain endothelium (AAV-BR1) combined with the CRISPR/Cas9 system (AAV-BR1-CRISPR) to specifically knock out genes of interest in brain ECs of adult mice. We first generated a mouse model expressing Cas9 in ECs (*Tie2*^Cas9^). We selected endothelial β-catenin (*Ctnnb1*) gene, which is essential for maintaining adult BBB integrity, as the target gene. After intravenous injection of AAV-BR1-sg*Ctnnb1*-tdTomato in 4-week-old *Tie2*^Cas9^ transgenic mice resulted in mutation of 36.1% of the *Ctnnb1* alleles, thereby leading to a dramatic decrease in the level of CTNNB1 in brain ECs. Consequently, *Ctnnb1* gene editing in brain ECs resulted in BBB breakdown. Taken together, these results demonstrate that the AAV-BR1-CRISPR system is a useful tool for rapid identification of endothelial genes that regulate BBB integrity *in vivo*.

## Introduction

The blood-brain barrier (BBB) acts as a selective interface separating the central nervous system (CNS) from the periphery, and maintaining a healthy environment for development and homeostasis of the CNS. Specialized endothelial cells (ECs), which comprise continuous intercellular tight junctions, specific expression of a series of transport proteins, and an extremely low rate of transcytosis, are the core component of the BBB 1. These features allow the endothelium to tightly control both paracellular and transcellular transport of molecules and ions across the BBB 1, 2.

To date, little is known regarding the mechanisms underlying maintenance of adult BBB homeostasis. Using gene targeting animals, researchers have revealed the physiological functions of intracellular signaling pathways and brain endothelial enriched transporters, such as VEGF 3, SHH 4, PDGFB/PDGFRβ 5, TGF-β/Smad 6, Wnt 7, GLUT1 8, and MFSD2A 9 in cerebrovascular development and homeostasis maintenance. Recently, researchers have employed high-throughput sequencing to provide numerous clues that may better explain the unique properties of brain ECs 9-11. However, only a handful of the discovered genes have been experimentally validated for their physiological functions in BBB development and homeostasis maintenance, mainly due to limited model systems. Over the last 50 years, researchers developed different models to simulate the BBB, including *in vitro*, *in vivo*, *in situ*, *ex vivo* or *in silico*. Among all, *in vivo* models closely mimic the BBB features as they comprise all components of the physical and physiological BBB, which has played indispensable roles in establishing a causal link between genomic mutations and phenotypes during cerebrovascular development and in vascular/neurological disease 12. Meanwhile, generating genetically modified mice is a time-consuming process. Overall, there is a lack of a technical approach for rapid identification of endothelial genes essential for regulating adult BBB homeostasis *in vivo*.

Somatic genome editing, based on the clustered regularly interspaced short palindromic repeats (CRISPR)/CRISPR-associated (Cas) system, has been applied to rapidly characterize functional genes in various tissues 13-16. Elegant studies have recently shown that CRISPR/Cas9 can be used to edit endothelial genes *in vivo* 17, 18. Specifically, the researchers applied recombinant AAV serotype 1 (rAAV1)-mediated CRISPR/Cas9 to successfully edit the vascular endothelial growth factor receptor 2 (*Vegfr2*) genomic loci of ECs and disrupt angiogenesis in mouse models of laser induced choroid neovascularization and oxygen-induced retinopathy 17. Another group employed an adenovirus harboring single-guide RNAs (sgRNAs), targeting activin receptor-like kinase 1 (*Alk1*) together with AAV-VEGF, to successfully induce mutations in the *Alk1* gene in brain ECs and generated brain arteriovenous malformation in adult mice 18. Nevertheless, an extensive and in-depth evaluation of CRISPR/Cas9's efficiency in studying the function of brain endothelial genes in BBB integrity *in vivo* remains unknown.

In the present study, we evaluated the feasibility and efficiency of CRISPR/Cas9-mediated gene editing in brain ECs of postnatal mice. This proof-of-concept study targeted the endothelial cadherin-associated protein beta 1 (β-catenin), which is encoded by *Ctnnb1*. The essential role of endothelial β-catenin in maintenance of adult BBB integrity has been demonstrated 19. Overall, our results indicated that adeno-associated virus targeting brain endothelium (AAV-BR1)-mediated delivery of sgRNAs can efficiently induce *Ctnnb1* gene disruption in brain ECs, thereby causing BBB breakdown in adult EC-restricted Cas9-knockin mice.

## Results

### Design and *in vitro* testing of sgRNAs

**Figure 1:**
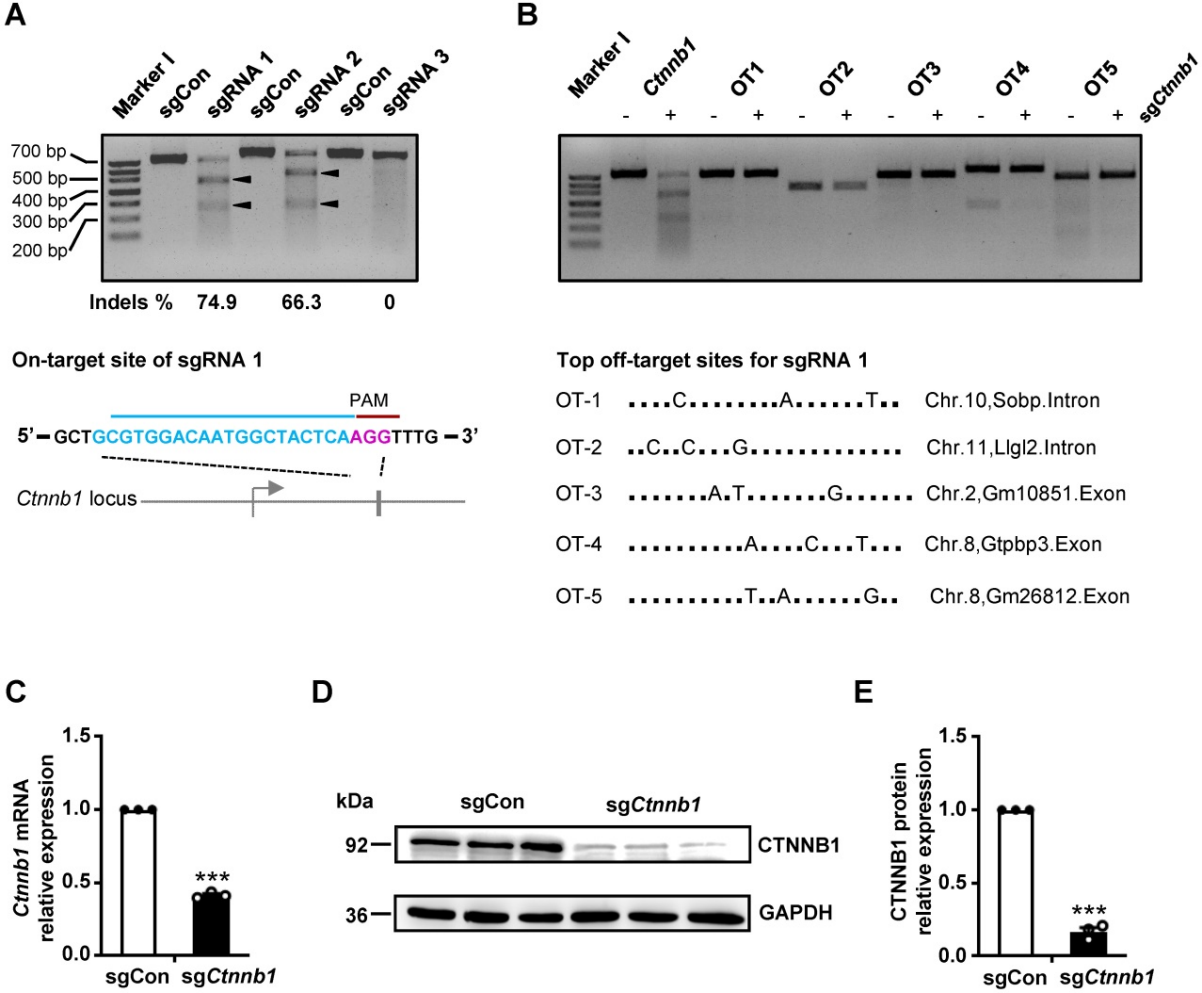
** sgRNA generation and *in vitro* gene editing using CRISPR/Cas9. (A)** T7E1 analysis on target sites of PCR-amplified genomic DNA from NIH-3T3 cells after transfection with either a control sgRNA (herein refered to as sgCon) or candidate sgRNAs (upper panel) and the schematic representation of the mouse *Ctnnb1* locus for sgRNA1 (hereafter refered to as sg*Ctnnb1*) (lower panel). Arrowheads indicate cleavage products for each sgRNA. Lane I was loaded with a molecular weight marker (100 bp ladder). PAM sequence marked in magenta. **(B)** T7E1 analysis of the top 5 potential OT DNA cleavage sites of PCR-amplified genomic DNA from NIH-3T3 cells after transfection with sg*Ctnnb1* (upper panel) and the schematic representation of the top 5 potential OT locus for sg*Ctnnb1* (lower panel). Lane I was loaded with a molecular weight marker (100 bp ladder). **(C)** qRT-PCR was performed to quantify mRNA expression in NIH-3T3 cells transfected with sgCon or sg*Ctnnb1*. Data presented are means ± stanadard error of the mean (SEM). (*n* = 3 for each sample, *** *P* < 0.001, two-tailed unpaired *t*-test). **(D and E)** Western blots **(D)** and quantification **(E)** of CTNNB1 protein levels in NIH-3T3 cells transfected with sgCon or sg*Ctnnb1*. Data are means ± SEM. (*n* = 3 for each sample, *** *P* < 0.001, two-tailed unpaired *t*-test).

To validate our CRISPR/Cas9 gene editing system, targeting brain ECs *in vivo*, we designed 3 sgRNAs against *Ctnnb1* using the CHOPCHOP CRISPR design tool 20. The 3 sgRNAs (*Ctnnb1*-sgRNA1-3) targeting different regions of exon 1 or exon 2 near the ATG of *Ctnnb1* were cloned into a Cas9-expressing vector, then transfected into NIH-3T3 cells. Next, we performed a T7 endonuclease 1 (T7E1) assay to evaluate targeting efficiency of the sgRNAs. Subsequently, we used PCR to amplify a ~700 bp DNA fragment of the targeted locus, then subjected it to T7E1 assay. The mutant *Ctnnb1* gene was digested into two smaller fragments (approximately 450 and 250 bp). Results showed that the *Ctnnb1*-sgRNA1 had the highest targeting efficiency, inducing 74.9% insertions and deletions (indels) (Fig. 1A). We did not detect any off-target (OT) DNA cleavage by T7E1 at the top 5 predicted OT sites of *Ctnnb1*-sgRNA1 (Fig. 1B). To further evaluate efficiency of sgRNA1, we detected the effect of *Ctnnb1* disruption on transcriptional and translational levels in NIH-3T3 cells transfected with the sgRNA1-Cas9 plasmid. Results showed a marked downregulation of *Ctnnb1* mRNA and protein expression in groups of sg*Ctnnb1* relative to sgControl (sgCon) after quantitative real-time PCR (qRT-PCR) analysis (Fig. 1C) and Western blots (Fig. 1D-E). Consequently, we used *Ctnnb1*-sgRNA1 to verify efficiency of the AAV-CRISPR system for genome editing in brain ECs. Overall, these results affirmed the selected *Ctnnb1*-sgRNA1 as an effective candidate for further studies.

### *In vivo* genome editing of brain ECs using AAV-BR1-CRISPR system

To establish a technical platform for somatic genome editing in brain ECs, we first crossed endothelial-specific *Tie2-Cre* transgenic mice 21 with *Rosa26^Cas9-GFP^* mice 22 and generated *Tie2*^Cas9^ mice (Fig. S1A), whose Cas9 is mainly expressed in ECs and hematopoietic cells. And the Cre-negative littermates were used as controls. Results from immunofluorescent staining of brain sections revealed no significant differences in BBB permeability, capillary density, vascular diameters, and vascular branch points between *Tie2*^Cas9^ and control mice (Fig. S1B-E), indicating that the expression of Cas9 in ECs did not affect cerebrovascular development.

**Figure 2:**
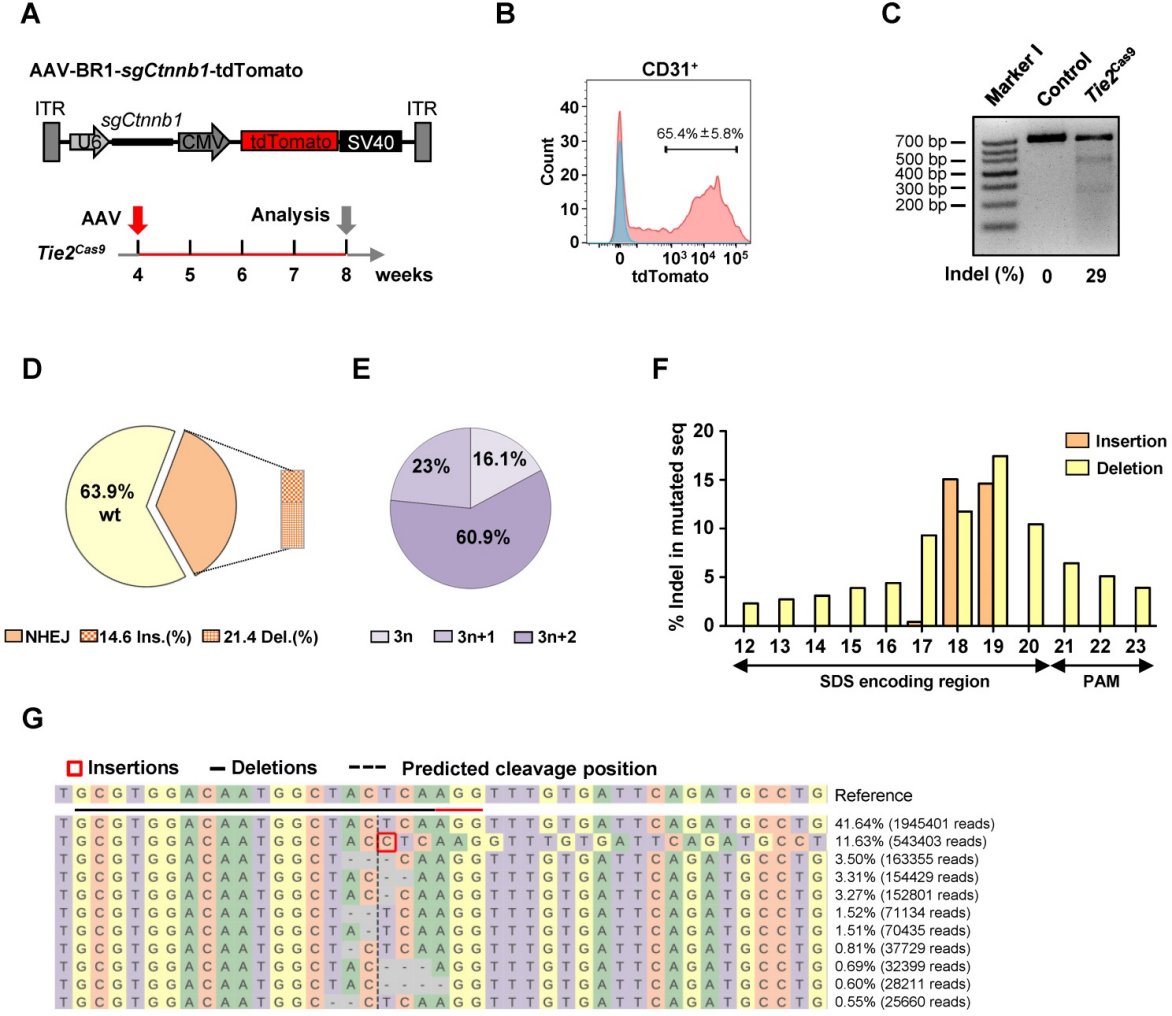
***In vivo* genome editing of brain ECs using the AAV-BR1-CRISPR system. (A)** The strategy used to incorporate the sg*Ctnnb1* vector into AAV-BR1 and the scheme of study. **(B)** Results of flow cytometry analysis used to detect CD31^+^tdTomato^+^ ECs of *Tie2*^Cas9^ mice with (red) or without (blue) AAV-BR1-sg*Ctnnb1*-tdTomato intravenous injection. Data are means ± SEM. (*n* = 4 mice). **(C)** T7E1 assay of brain ECs isolated from *Tie2*^Cas9^ mice at the targeted locus of *Ctnnb1* compared to the control group. Lane I was loaded with a molecular weight marker (100 bp ladder). **(D)** Classification of amplicon sequencing of brain ECs at the *Ctnnb1* locus. (*n* = 3 mice). **(E)** Indel phase showing that most indels caused a frameshift. (*n* = 3 mice). **(F)** Proportion of sequences containing specific mutation types (insertions or deletions) at individual base pair positions out of all mutated sequences. (*n* = 3 mice). **(G)** Summary of the most abundant indels in brain ECs obtained from *Tie2*^Cas9^ mice treated with AAV-BR1-sg*Ctnnb1*-tdTomato based on CRISPResso2 analysis. Red rectangles denote inserted sequences, while dashes represent deleted nucleotides. The horizontal dashed line indicates the CRISPR cut site.

Our previous work, and findings from other labs, have shown that AAV-BR1, a brain microvasculature EC-specific viral vector modified from AAV2, has a high specificity and long-term transduction efficiency for brain ECs 23, 24. Therefore, we cloned the *Ctnnb1*-sgRNA1 (hereafter referred to as sg*Ctnnb1*) and tdTomato into a U6 and CMV-driven AAV backbone, then packaged it into AAV-BR1 (AAV-BR1-sg*Ctnnb1*-tdTomato) (Fig. 2A). Control and *Tie2*^Cas9^ mice were intravenously injected with a single dose of 1.8 × 10^11^ vg (viral genomes) of AAV-BR1-sg*Ctnnb1*-tdTomato at 30 days postnatal (P30), then analyzed 4 weeks later (P60) (Fig. 2A). We did not detect tdTomato expression in liver, spleen, kidney, stomach, and heart (Fig. S2A-F), which was consistent with a previous report 24. These results confirmed that AAV-BR1-sg*Ctnnb1*-tdTomato did not transduce the ECs of organs other than the brain. Next, we performed a T7E1 assay to confirm that the *Ctnnb1* gene was not edited in the lung, spinal cord, liver, and kidney (Fig. S3A). After injection of AAV-BR1-sg*Ctnnb1*-tdTomato, we isolated primary brain ECs from control and *Tie2*^Cas9^ mice by fluorescence-activated cell sorter (FACS), and found that ~65% brain ECs were successfully transduced by AAV (CD31 and tdTomato double-positive cells) (Fig. 2B). T7E1 analysis of PCR products of 3 mice amplified from the genomic loci of *Ctnnb1* gene of brain ECs revealed that ~29% of the *Ctnnb1* genomic loci were efficiently edited in *Tie2*^Cas9^ mice transduced by AAV-BR1-sg*Ctnnb1*-tdTomato (Fig. 2C). In addition, no OT DNA cleavages were detected in the top 5 predicted OT sites in the genome of *Tie2*^Cas9^ mice (Fig. S3B). To ascertain efficiency of genome editing in brain ECs, we performed targeted deep sequencing targeting the *Ctnnb1* genomic locus in brain ECs isolated from the *Tie2*^Cas9^ mice transduced by AAV-BR1-sg*Ctnnb1*-tdTomato. Results showed that ~36.1% of the *Ctnnb1* genomic loci were mutated in *Tie2*^Cas9^ mice transduced by AAV-BR1-sg*Ctnnb1*-tdTomato (Fig. 2D), of which ~83.9% were out-of-frame (3n+1 or 3n+2) mutations (Fig. 2E). We also recorded high deletion and insertion rates in the *Ctnnb1* genomic locus, matching nucleotide positions 17 to 19 of sg*Ctnnb1* (Fig. 2F). Results from CRISPResso2 analysis revealed that most indel mutations were close to the predicted cleavage site of the *Ctnnb1* genomic locus (Fig. 2G). Taken together, these results indicated that systemic delivery of sgRNA via AAV-BR1 induced efficient genome editing in brain ECs of *Tie2*^Cas9^ mice.

### Genome editing decreased *Ctnnb1* expression and caused BBB breakdown in brain ECs of *Tie2*^Cas9^ mice

**Figure 3:**
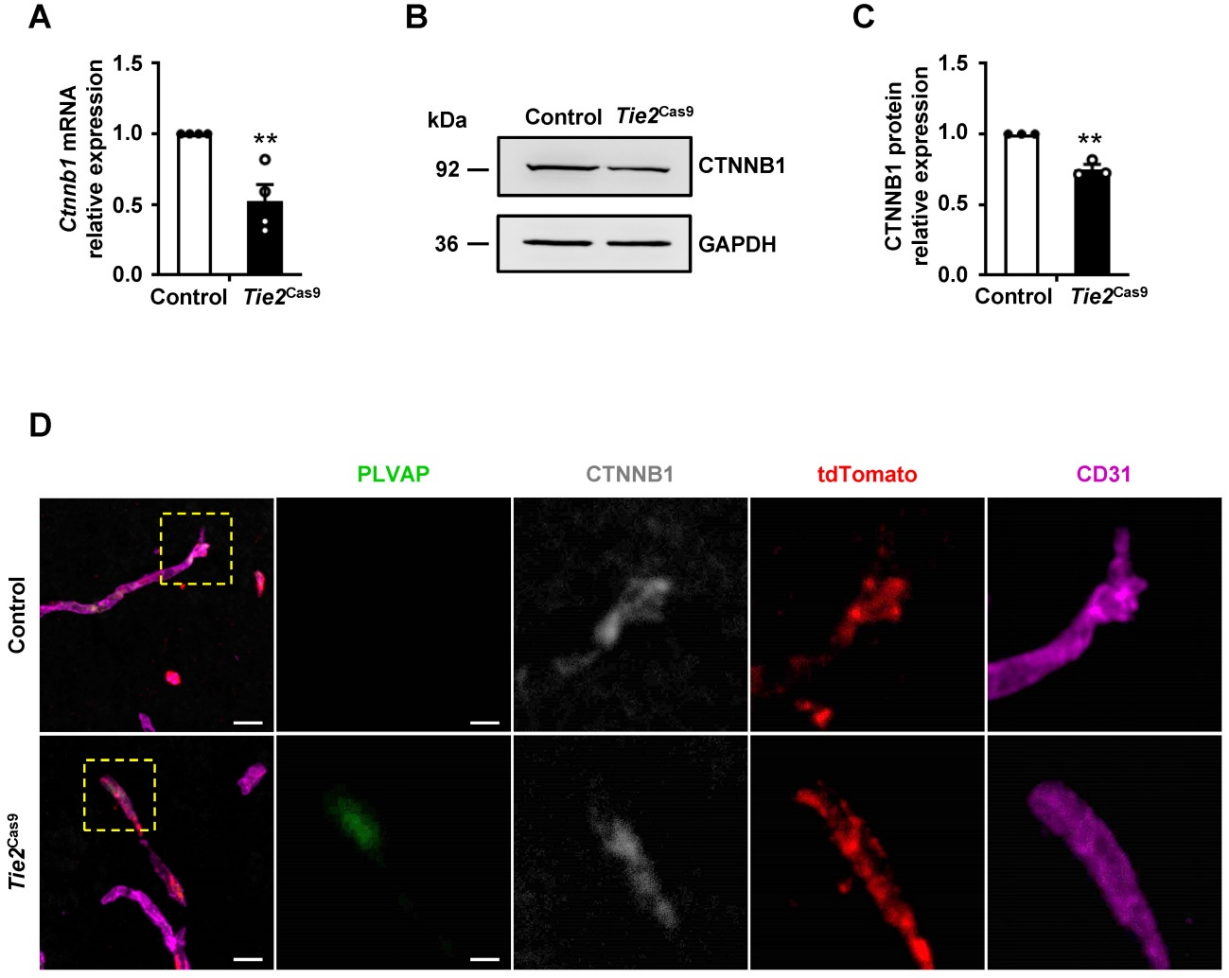
***In vivo* genome editing decreased CTNNB1 expression. (A)** qRT-PCR analysis of *Ctnnb1* mRNA expression in *Tie2*^Cas9^ mice treated with AAV-BR1-sg*Ctnnb1*-tdTomato. Data are means ± SEM. (*n* = 4 mice, ** *P* < 0.01, two-tailed unpaired *t*-test). **(B and C)** Western blots (B) and quantification (C) of CTNNB1 levels in brain ECs isolated from edited mice. Data are means ± SEM. (*n* = 3 mice, ** *P* < 0.01, two-tailed unpaired *t*-test). **(D)** Confocal microscopy images of PLVAP (green), CTNNB1 (gray), tdTomato (red), and CD31 (purple) immunostaining in *Tie2*^Cas9^ mice. Scale bar, 10 µm (left), 4 µm (right).

Next, we analyzed expression levels of *Ctnnb1* mRNA and protein in brain ECs isolated from control and *Tie2*^Cas9^ mice transduced by AAV-BR1-sg*Ctnnb1*-tdTomato. Brain CD31^+^7AAD^-^ ECs of control and *Tie2*^Cas9^ mice were sorted by FACS. The mRNA and protein was extracted from 3 mice for further experiment. Results revealed a ~40% downregulation of mRNA transcripts in sg*Ctnnb1*-edited *Tie2*^Cas9^ mice relative to controls (Fig. 3A). Western blots revealed approximately 25% reduction in CTNNB1 levels in sg*Ctnnb1*-edited* Tie2*^Cas9^ mice (Fig. 3B-C). Next, we used immunostaining to further detect CTNNB1 levels in control and *Tie2*^Cas9^ mice transduced by AAV-BR1-sg*Ctnnb1*-tdTomato and found that CTNNB1 levels were decreased in tdTomato-positive ECs of *Tie2*^Cas9^ mice in contrast to control counterparts (Fig. 3D). Previous studies have shown that inactivation of the *Ctnnb1* gene causes upregulation of plasma vesicle-associated protein (PLVAP), a protein associated with trans-endothelial transport and BBB function 7, 19, 25. As expected, PLVAP was upregulated in ECs deficient in *Ctnnb1* expression in the *Tie2*^Cas9^ mice transduced by AAV-BR1-sg*Ctnnb1*-tdTomato relative to controls (Fig. 3D). However, PLVAP was not detected in ECs maintaining CTNNB1 level, indicating a mosaic pattern of *Ctnnb1* gene disruption in the edited *Tie2*^Cas9^ mice (Fig. 3D). Previous studies have also shown that the tight junction protein *Claudin-5* gene is upregulated by the Wnt/β-catenin signaling pathway, which is opposite to how *Plvap* is regulated 7, 25. Results from immunostaining showed markedly lower levels of CLAUDIN-5 in the brain ECs from *Tie2*^Cas9^ mice transduced by AAV-BR1-sg*Ctnnb1*-tdTomato relative to controls, whereas PLVAP was significantly upregulated (Fig. S3D). Taken together, these results demonstrated that AAV-BR1-sg*Ctnnb1*-tdTomato induced successful disruption of the *Ctnnb1* gene in brain ECs of *Tie2*^Cas9^ mice.

**Figure 4:**
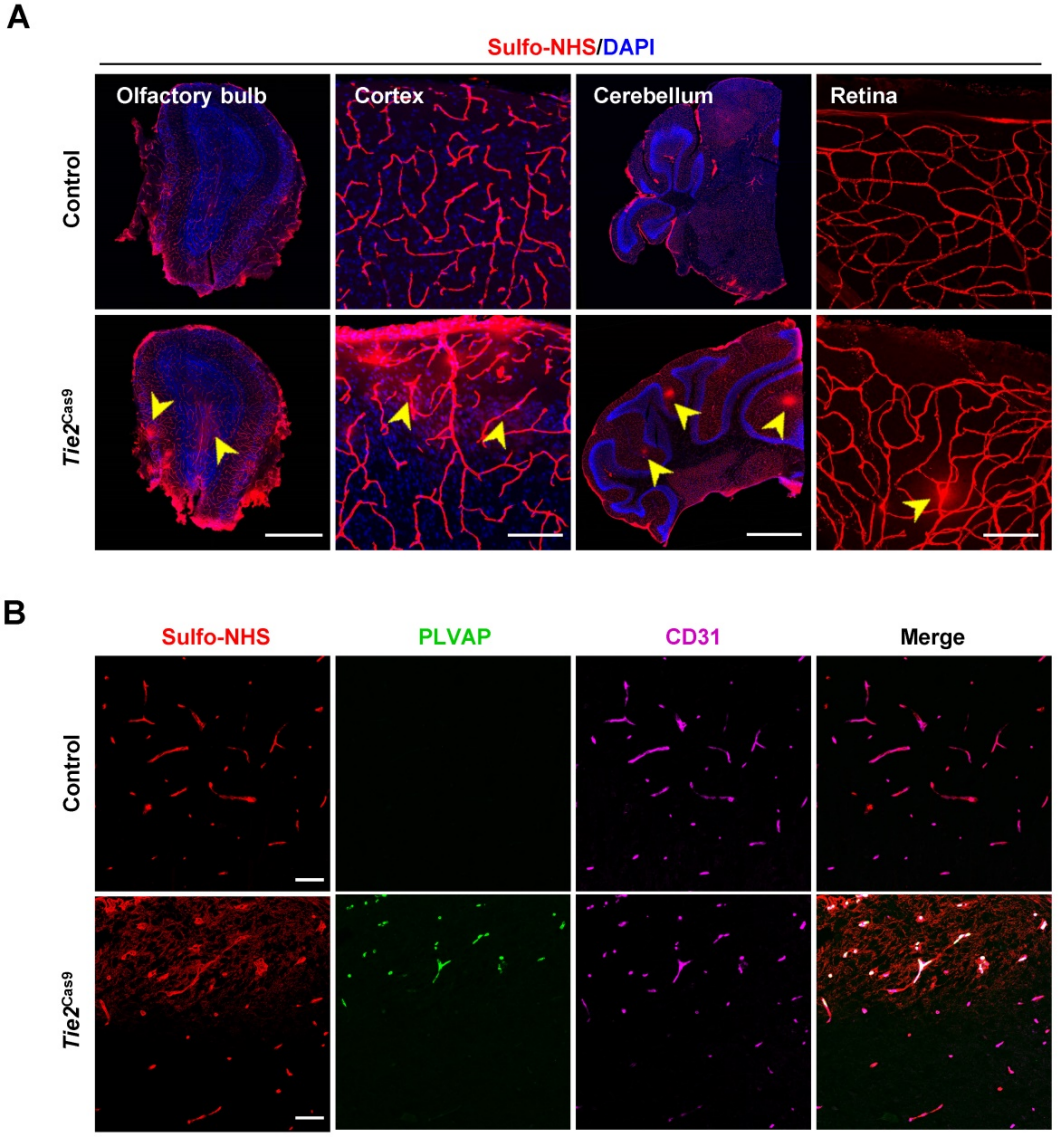
***In vivo* genome editing of the *Ctnnb1* gene causes BBB breakdown. (A)** Sulfo-NHS-LC-Biotin tracer (red) injection revealed BBB/BRB defects in the olfactory bulb, cerebral cortex, cerebellum, and retina in *Tie2*^Cas9^ mice at P60 (4 weeks post- AAV-BR1-sg*Ctnnb1*-tdTomato treatment). Scale bar, 500 µm (olfactory bulb), 100 µm (cerebral cortex), 1 mm (cerebellum), 100 µm (retina). **(B)** *Tie2*^Cas9^ mice at P60 (4 weeks post-AAV-BR1-sg*Ctnnb1*-tdTomato treatment) showed PLVAP induction in the Sulfo-NHS-LC-Biotin leakage region. Scale bar, 50 µm.

To examine the consequence of editing the *Ctnnb1* gene, we detected BBB permeability in *Tie2*^Cas9^ mice treated with AAV-BR1-sg*Ctnnb1*-tdTomato relative to controls. Analysis by leakage of Sulfo-NHS-LC-Biotin from the intravascular space to the parenchyma revealed widespread BBB leakage in the olfactory bulb, cortex, cerebellum, and retina of *Tie2*^Cas9^ mice transduced by AAV-BR1-sg*Ctnnb1*-tdTomato (Fig. 4A). Notably, this leakage mainly originated from the tdTomato positive area (Fig. S3C). PLVAP, a permeability-associated protein, is associated with high vesicular transport and abnormal development of the BBB. During early development, *Plvap* is initially expressed in immature brain vessels, then later strongly downregulated in ECs of BBB 26. Conversely, *Plvap* is strongly upregulated under the pathological condition of the BBB 27. Based on this, we performed an immunostaining assay to examine levels of PLVAP expression in *Tie2*^Cas9^ mice, and found significant upregulation in the Sulfo-NHS-LC-Biotin leakage area (Fig. 4B). Taken together, these results confirmed successful establishment of a BBB disruption mouse model via the AAV-BR1-CRISPR system.

## Discussion

In this study, we successfully established an AAV-BR1-CRISPR system by combining AAV-BR1-mediated mosaic transduction and somatic mutagenesis based on CRISPR/Cas9. This system can effectively and specifically achieve genome editing in brain ECs postnatal. This is the first report evaluating efficiency of the CRISPR/Cas9 system in brain ECs. Notably, systematic delivery of AAV-BR1-CRISPR effectively led to ~36.1% endothelial *Ctnnb1* gene editing, while our results also demonstrated that AAV-BR1-CRISPR mediated genome editing of brain endothelial *Ctnnb1* gene is sufficient for induction of BBB breakdown *in vivo*. Taken together, these results indicate that the AAV-BR1-CRISPR system has potential for future *in vivo* genetic screening of key regulators that regulate development and maintenance of brain vasculature, and is also expected to guide elucidation of the function of brain endothelial genes in various physiological or pathological processes, such as BBB breakdown. When combined with single-cell phenotyping, the ability of the AAV-BR1-CRISPR system to generate mosaic gene mutations is expected to greatly help in defining the cell-autonomous functions of endothelial genes.

Our results demonstrated that the BBB breakdown mouse model generated by AAV-BR1-CRISPR mediated *Ctnnb1* genome editing mimics the BBB phenotypes of traditional *Ctnnb1* knockout mice. However, sg*Ctnnb1*-edited* Tie2*^Cas9^ mice did not develop petechial hemorrhages and ataxia (data not shown) as the adult inducible EC-restricted *Ctnnb1* knockout mice 28. We considered that was because *Ctnnb1* was just decreased instead of completely deleted. Notably, AAV-BR1-CRISPR-mediated disruption of the *Ctnnb1* gene upregulated and downregulated PLVAP and CLAUDIN-5 expression, respectively, thereby resulting in BBB breakdown. This indicates that the AAV-BR1-CRISPR system can serve as an alternative approach for generating mouse disease models associated with the dysfunction of brain endothelial genes.

In summary, we successfully developed an AAV-BR1-CRISPR system for rapid and efficient gene inactivation of postnatal brain ECs. This platform is not only suited for *in vivo* genetic screening of functional endothelial genes, but is also useful for generating mouse models of human diseases. Considering that the occurrence and development of neurological diseases, such as Alzheimer's disease and Parkinson's syndrome are related to cerebrovascular abnormalities 29, we expect that the AAV-BR1-CRISPR system will be widely used for dissecting the vascular mechanisms underlying neurological diseases.

## Materials and Methods

### Mice

All animal experiments were performed using protocols approved by the Animal Experiment Committee of the Beijing Institute of Lifeomics. EC-specific Cas9 gene knock-in mice (*Tie2*^Cas9^) were obtained by crossing previously reported *Tie2*-Cre transgenic mice 21 with their Cas9 gene knock-in counterparts 22, with Cre-negative littermates used as controls. Only male mice, at least 3 per genotype, were used in the experiments. The animals were bred under specific pathogen-free conditions. All mice were bred in a C57BL/6 background.

### Cell lines

Mouse embryonic fibroblast (NIH-3T3) cells (ATCC, CRL-1658) were cultured in DMEM (Biological Industries, 06-1055-57-1ACS), supplemented with 10% FBS (GIBCO, 10099-141) and 1% antibiotic/antimycotic solution (GIBCO, 15240-062), and incubated at 37°C under 5% CO_2_. Human embryonic kidney (HEK293T) cells (ATCC, CRL-11268) were cultured in DMEM, supplemented with 10% FBS (10099-141, Gibco) and 1% antibiotic/antimycotic solution (GIBCO, 15240-062) and maintained at 37°C with 5% CO_2_.

### sgRNA constructs and transfection

The candidate sgRNA sequences were annealed and cloned into a PX459 plasmid (Addgene plasmid, 48139), after restriction digestion with Esp3I (Thermo Fisher Scientific, FD0454). PX459 vectors with candidate sgRNAs were then seeded in 6-well plates and transfected into NIH-3T3 cells (at 60% confluence) using Lipofectamine™ 3000 (Invitrogen, L3000075) according to the manufacturer's protocol. The transfected cells were selected using Puromycine (2.5 µg/mL) prior to use for subsequent studies.

### T7E1 assay

Genomic DNA was isolated from the cells using a DNeasy Tissue Kit (Qiagen), then target sites for the *Ctnnb1* and OT genes amplified using KOD DNA polymerase (Toyobo, KFX-201) according to the manufacturer's protocol. A list of primers for the target genes is outlined in Supplementary Table. PCR products were denatured and annealed using a thermal cycler as follows: the PCR product was first mixed with 2 µL of NEBuffer 2 to a total volume of 19 µL to run a reannealing process to enable heteroduplex formation: the reaction mixture was then subjected to 95°C for 5 minutes, ramped down to 85°C at a rate of -2°C/s, then ramped down to 25°C at a rate of -0.1°C/s. Annealed PCR products were incubated with 1 µL T7E1 enzyme (NEB, M0302S) for 25 minutes at 37°C, then confirmed via agarose gel electrophoresis. The experiments were repeated 3 times.

### AAV-BR1 production

Brain microvascular EC specific AAV-BR1 viral vector was modified from AAV2, generously provided by Prof. Jakob Korbelin 24. The sgRNA was cloned into an AAV vector containing terminal repeats of AAV2 and the SV40 Poly-A sequence. Thereafter, a CMV promoter flowing 2A-tdTomato was cloned before SV40 Poly-A of the AAV vector to generate the plasmid and pAAV-sg*Ctnnb1*-CMV-tdTomato. All viruses were generated by the triple transfection method with AAV package plasmid, transgene plasmid and helper plasmid (Cell Biolabs), using HEK293T cells. Cells were subjected to high-speed centrifugation, at day 3 post transfection, and the supernatant and cell fragments collected. Pure vectors were separated and extracted, after one round of ultracentrifugation, then desalted using an Amicon Ultra-Centrifugal Filter device (EMD Millipore). The obtained viruses were injected into the tail vein of mice at a dose of 1.8 ×10^11^ vg per mouse.

### Flow cytometry

Brain were dissected from mice after AAV-BR1 treatment, then digested in PBS containing 0.2% collagenase H (Roch, 33278626) and 10% BSA (Sigma, WXBD0126 V) at 37 °C for 1 hour. The contents were centrifuged, lipids cleared, then samples incubated with CD31-APC antibodies (eBioscience, MA110191) and processed for FACS analysis according to the manufacturer's procedures. We excluded dead cells by 7-amino-actinomycin D staining.

### RNA extraction and qRT-PCR

Total RNA was extracted using the TRIzol Reagent (Life Technologies, 15596026), according to the manufacturer's instructions, then reverse transcribed into cDNA using reverse transcriptase master mix (TOYOBO, FSQ-201). The cDNA was subjected to qRT-PCR, performed on a QuantStudio 3 Real-time PCR system (Applied Biosystems) using the SYBR Green Real-time PCR Master Mix (TOYOBO, QPK-201) according to the manufacturer's instructions. The oligos for target genes used in qRT-PCR are listed in the Supplementary Table. The experiments were repeated 4 times.

### Western blot analysis

Brain ECs (CD31^+^7AAD^-^) were isolated by FACS and lysed in RIPA supplemented with protease inhibitors (Roche) to obtain total proteins. Protein lysates were quantified using the Pierce BCA Protein Assay Reagent (Thermo Fisher Scientific, 23225), and then were separated using 10% sodium dodecyl sulphate polyacrylamide (SDS-PAGE) gels, and transferred to PVDF membrane (Millipore). The membrane was blocked with 5% milk for 1 hour, then incubated overnight at 4°C with the following primary antibodies: rabbit anti-β-catenin (1:1000, Cell Signaling Technology, D10A8) and GAPDH (1:1000, Zsbio, TA-08). The membranes were then incubated with horseradish peroxidase-conjugated secondary antibodies and developed using enhanced chemiluminescent (ECL). The experiments were repeated 3 times.

### Immunostaining

Brain tissues were first harvested and fixed overnight in 4% paraformaldehyde (PFA). Thereafter, the tissues were either embedded in paraffin and sectioned at 6 µm, or sectioned at 40 µm in OCT at -30 °C (SAKURA). The sections were blocked with 10% goat serum for 30 minutes at 37 °C, then incubated with the following primary antibodies overnight at 4 °C: CD31 (1:100, BD Biosciences, 550274), GFP (1:500, Cell Signaling Technology, 2956s), RFP (1:500, Rockland, Limerick, PA, 600-401-379; tdTomato can be recognized by the RFP antibody), β-catenin (1:300, Cell Signaling Technology, 8480) PLVAP/MECA-32 (1:200, BD PharMingen, 553849), CLAUDIN-5 (1:500, Invitrogen, 352588). The samples were then incubated with the corresponding Alexa Fluor-conjugated secondary antibodies (1:1000, Thermo Fisher Scientific), mounted with ProLong Gold (Invitrogen) then subjected to confocal microscopy on the LSM 880 microscope (Carl Zeiss AG).

### BBB permeability assay

Deeply anesthetized mice were intravenously injected with Sulfo-NHS-LC-Biotin (0.5 mg/g body weight, Thermo Fisher Scientific, 21335) in saline. Their brain tissues were dissected out, after 5 minutes of circulation, fixed in 4% PFA overnight, equilibrated in 30% sucrose, then frozen in TissueTek OCT. The tissues were sectioned (40 µm), fixed in 4% PFA for 15 minutes at room temperature, then incubated with corresponding Alexa Fluor-594-conjugated secondary antibodies. Samples were analyzed by confocal microscopy (LSM 880, Carl Zeiss AG).

### Deep sequencing

Target regions were amplified from genomic DNA by Taq DNA polymerase (Toyobo, KFX-201), and the PCR products subjected to paired-end sequencing on the Illumina MiSeq platform at GENEWIZ, Inc. (Suzhou, China). Raw reads were cleaned and analyzed using CRISPResso2. Indels in the reads were compared with the reference and centered on the Cas9 predicted cleavage site from CRISPResso2, which describes the length and position of the indels in the alignment.

### Statistics

Data were analyzed using GraphPad Prism 8 software. Data were analyzed using a two-tailed unpaired Student's *t*-test. The error bars on graphs represent the mean ± standard deviation (SD). * *P* < 0.05, ** *P* < 0.01 and *** *P* < 0.001 were considered statistically significant.

## Supplementary Material

Supplementary figures and table.

## Abbreviations

EC: endothelial cell
BBB: blood-brain barrier
CNS: central nervous system
CRISPR: clustered regularly interspaced short palindromic repeats
*Vegfr2*: vascular endothelial growth factor receptor 2
sgRNAs: single-guide RNAs
*Alk1*: activin receptor-like kinase 1
*Ctnnb1*: cadherin associated protein beta 1
PAM: protospacer adjacent motif
T7E1: T7 endonuclease 1
OT: off-target
Indel: insertion and deletion
FACS: fluorescence-activated cell sorter
*Plvap*: plasma vesicle-associated protein
PFA: paraformaldehyde
