# Supplementary material for: Genome Editing with AAV-BR1-CRISPR in Postnatal Mouse Brain Endothelial Cells
Source: Int J Biol Sci. 2022 Jan 1;18(2):652–60. doi: 10.7150/ijbs.64188 (PMC8741854; doi:10.7150/ijbs.64188)
Supplement: Supplementary file 1 — Supplementary figures and table. [file ijbsv18p0652s1.pdf]

Supplementary Figures:

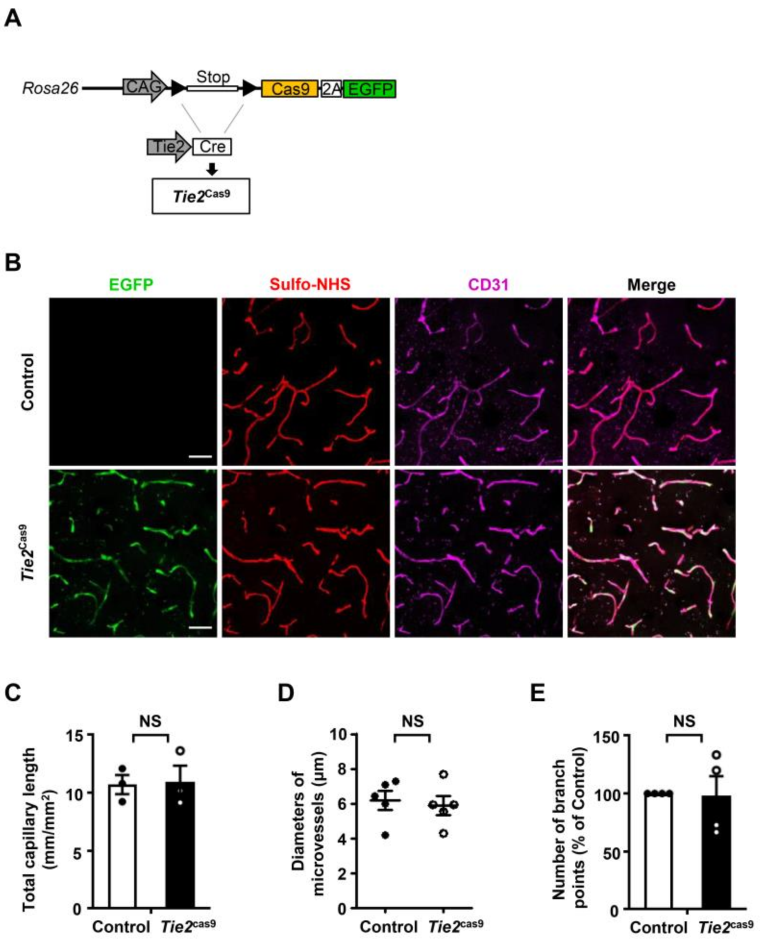

Figure S1. EC-specific Cas9-expressing mouse exhibited a normal brain vasculature.

(A) The strategy used for generating *Tie2*^{Cas9} mice. (B) Sulfo-NHS-LC-Biotin tracer extravasation assay revealed no significant differences in BBB integrity in brain sections of control and *Tie2*^{Cas9} mice. Scale bar, 40 μm. (C-E) Quantification of the cerebral capillary length (*n* = 3 mice), microvessel diameters (*n* = 5 mice), and branch points (*n* = 4 mice), showing a comparable brain vasculature in control and *Tie2*^{Cas9} mice. Data are means ± SEM. (NS = not significant, two-tailed unpaired *t*-test).

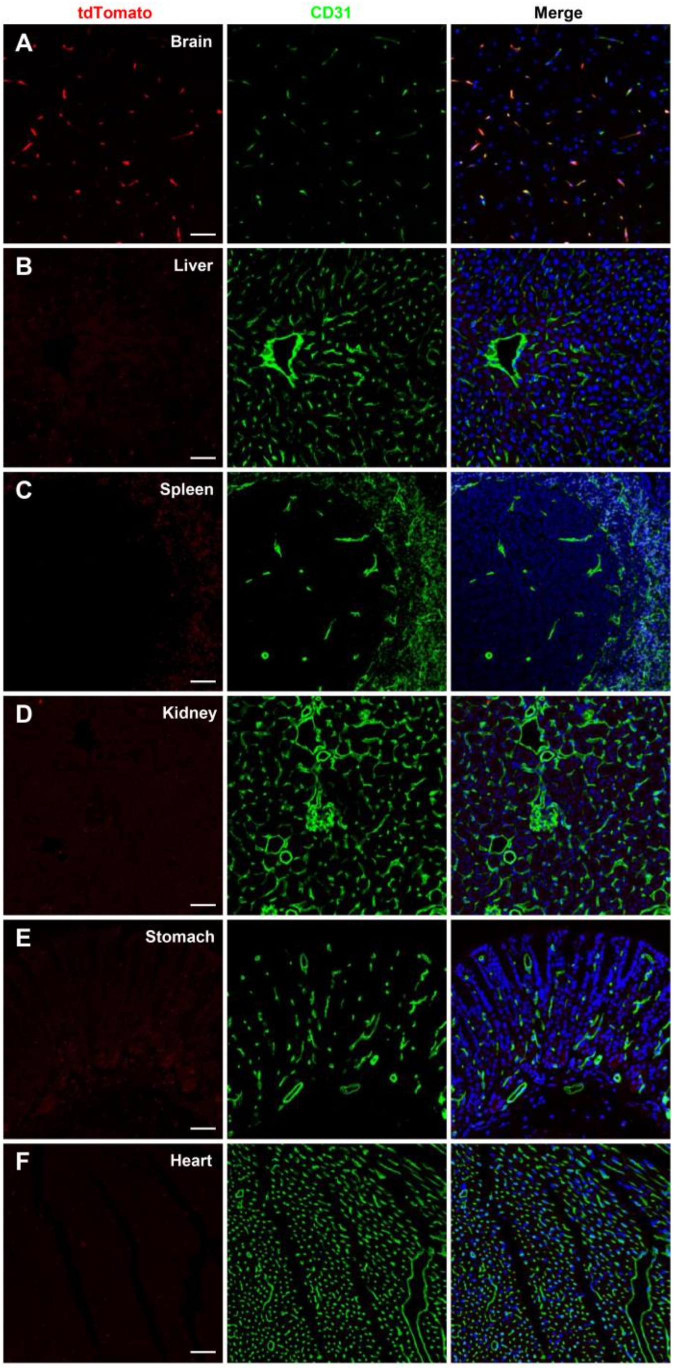

Figure S2. AAV-BR1 specifically transduces ECs in brain.

(A-F) Immunostaining of tdTomato (red), CD31 (green) and DAPI (blue) from brain (A), liver (B), spleen (C), kidney (D), stomach (E) and heart (F) 4 weeks after vector injection, showing that AAV-BR1-tdTomato specifically transduces the brain ECs. Scale bar, 50 μm.

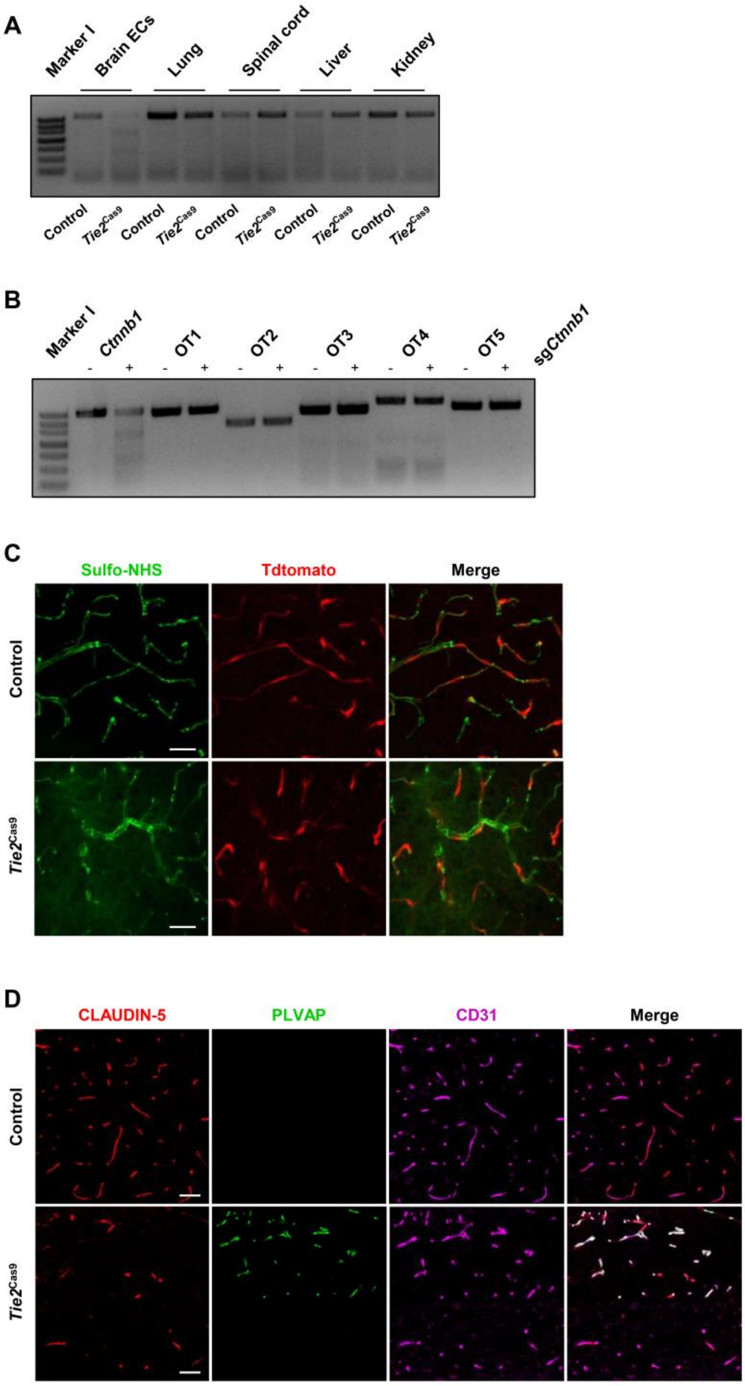

Figure S3. ***In vivo*** **genome editing of brain ECs using AAV-BR1-CRISPR system.**

(A) T7E1 analysis on target sites of PCR-amplified genomic DNA in lungs, spinal cord, liver, and kidney from control and *Tie2*^{Cas9} mice 4 weeks after AAV-BR1-sg*Ctnnb1*-tdTomato injection. Lane I was loaded with a molecular weight marker (100 bp ladder). (B) T7E1

analysis of the top 5 potential OT sites of PCR-amplified genomic DNA of brain ECs isolated from *Tie2*^{Cas9} mice 4 weeks after AAV-BR1-sg*Ctnnb1*-tdTomato injection. Lane I was loaded with a molecular weight marker (100 bp ladder). (C) Sulfo-NHS-LC-Biotin tracer (green) injection generated BBB defects, mainly in tdTomato positive area. Scale bar, 30 μm. (D) Immunostaining of CLAUDIN-5 (red), PLVAP (green) and CD31 (purple) showing downregulation of CLAUDIN-5, and upregulation of PLVAP in *Tie2*^{Cas9} mice at P60 (4 weeks after AAV-BR1-sg*Ctnnb1*-tdTomato treatment). Scale bar, 50 μm.

Supplementary Table:

SgRNA oligos and primers for genotyping, T7E1 analysis, DNA sequencing, and qRT-PCR analysis.

**Name**	**Sequence(5'-3')**	**Purpose**
Tie2 Cre fwd	GAAGGGCAAGATGGATAGGGC	mouse genotyping
Tie2 Cre rev	GCATCGACCGGTAATGCAGGC	mouse genotyping
Cas9 Tg-1	GAGGCAGGAAGCACTTGCTCT	mouse genotyping
Cas9 Tg-2	TGGCGTTACTATGGGAACATACG	mouse genotyping
Cas9 Tg-3	TACACCTGTTCAATTCCCCTGC	mouse genotyping
sgCON fwd	CACCGAGCTCGCCATGTCGGTTCTC	*In vitro* sgRNA
sgCON rev	CGTGAACCGACATGGCGAGCTCAAA	*In vitro* sgRNA
Ctnnb1 sgRNA1 fwd	CACCGGCGTGGACAATGGCTACTCA	*In vitro* and *in vivo* sgRNA
Ctnnb1 sgRNA1 rev	CTGAGTAGCCATTGTCCACGCCAAA	*In vitro* and *in vivo* sgRNA
Ctnnb1 sgRNA2 fwd	CACCGTGACCTGATGGAGTTGGACA	*In vitro* sgRNA

Ctnnb1 sgRNA2 rev	CTGTCCAACTCCATCAGGTCACAAA	*In vitro* sgRNA
Ctnnb1 sgRNA3 fwd	CACCGGTTGGACATGGCCATGGAGC	*In vitro* sgRNA
Ctnnb1 sgRNA3 rev	CGCTCCATGGCCATGTCCAACCAAA	*In vitro* sgRNA
sgRNA1 fwd	AGCACCGTATGCCTACAATCT	T7E1 primer
sgRNA1 rev	CATCTTCTTCCTCAGGGTTGC	T7E1 primer
sgRNA 2 fwd	CTCTTCCCTTCTGCACACTAC	T7E1 primer
sgRNA 2 rev	GGACATTAGTGGGATGAGCA	T7E1 primer
sgRNA 3 fwd	CTCTTCCCTTCTGCACACTAC	T7E1 primer
sgRNA 3 rev	GGACATTAGTGGGATGAGCA	T7E1 primer
Sobp fwd	GTGAGACGACTAGACACGCTTAG	*Ctnnb1* OT1 T7E1
Sobp rev	GCCTGTCTATCTTATTGCTTTAGC	*Ctnnb1* OT1 T7E1
Llgl2 fwd	CCTACATTGACTTCGGAGAGG	*Ctnnb1* OT2 T7E1
Llgl2 rev	GTCAGGGACAATGTGATTAAGAAC	*Ctnnb1* OT2 T7E1
Gm10851 fwd	CACATACACACATGAGCACATG	*Ctnnb1* OT3 T7E1
Gm10851 rev	CTCAGTGAGGGACTGTCTGAC	*Ctnnb1* OT3 T7E1
Gtpbp3 fwd	CATGAGTCACAGCTGTATTCACTT	*Ctnnb1* OT4 T7E1
Gtpbp3 rev	CTCTAGATCAACTGAGCAAAGCT	*Ctnnb1* OT4 T7E1
Gm26812 fwd	GAGTATTGACCTAGCACCTGATAG	*Ctnnb1* OT5 T7E1
Gm26812 rev	CACGTGGTGAGAGCTGAATC	*Ctnnb1* OT5 T7E1
Ctnnb1 fwd	AGATCGGAAGAGCACACGTCTGAACTCCA<br>GTCACNNNN<br>GTATAGGTAGCAGAATCACGG	DNA sequencing

Ctnnb1 rev	TGCTGATTATTTCACCAAGCCNNNNAGATC GGAAGAGCGTCGTGTAGGGAAAGAGTG	DNA sequencing
Ctnnb1 exon 9 fwd	GGTGCTATTCCACGACT	qRT-PCR
Ctnnb1 exon 10 rev	CCCTTCTACTATCTCCTCC	qRT-PCR
GAPDH fwd	TGCCCAGAACATCATCCCT	qRT-PCR
GAPDH rev	GGTCCTCAGTGTAGCCCAAG	qRT-PCR

*NNNN = index from Illumina TruSeq Small RNA Sample Prep Kit
